# Significance of melting heat in bioconvection flow of micropolar nanofluid over an oscillating surface

**DOI:** 10.1038/s41598-023-38361-w

**Published:** 2023-07-20

**Authors:** M. S. Alqurashi, Umar Farooq, Mirwais Sediqmal, Hassan Waqas, Sobia Noreen, Muhammad Imran, Taseer Muhammad

**Affiliations:** 1 Department of Mathematics and Statistics, College of Science, Taif University, P.O. Box 11099, 21944 Taif, Saudi Arabia; 2 Department of Mathematics, Government College University Faisalabad, Faisalabad, 38000 Pakistan; 3 Department of Civil Engineering, Engineering Faculty, Laghman University, Mehtarlam, Laghman 2701 Afghanistan; 4 School of Energy and Power Engineering, Jiangsu University, Zhenjiang, 212013 China; 5 Department of Chemistry, Government College Women University Faisalabad, Faisalabad, 38000 Pakistan; 6 Department of Mathematics, College of Sciences, King Khalid University, 61413 Abha, Saudi Arabia

**Keywords:** Engineering, Mathematics and computing, Physics

## Abstract

Pharmaceuticals, biological polymer synthesis, eco-friendly uses, sustainable fuel cell innovations, microbial-enhanced extraction of petroleum, biological sensors, biological technology, and continual mathematical modeling refinement are all examples of how bioconvection is applied. This study examines the bio convectional viscoelastic-micropolar nano liquid flow with non-uniform heat sink/source, motile microorganisms that move across a stretched sheet. Thermal radiation and thermal conductivity are also explored. Brownian and thermophoresis diffusion effects are taken into account. The system of a higher partial differential equation is transformed to ODEs by using the appropriate similarity functions. Such reported equations are implemented with the computational tool MATLAB shooting approach using a bvp4c solver. The variations of numerous flow parameters comprise velocity, temperature, concentration, and motile microorganism profile. Various important, interesting transport numbers are numerically and graphically demonstrated with physical justifications. The bouncy ratio parameter reduces the fluid's velocity profile whereas the material parameter increases it. For increased melting parameters, the micro rotation profile improves, but it deteriorated. For the Prandtl number and temperature ratio parameters, the temperature profile is negative. The melting parameter influences the concentration profile. The microorganism’s profile is decreased bioconvective Lewis numbers and is higher for the magnetic parameter. The current model has many features in the manufacturing industries, engineering works, physics, and applied mathematics.

## Introduction

The study of heat and mass transfer in hydromagnetic flow with the magnetic effect of micropolar nanofluids over a stretched sheet has sparked a lot of interest due to its numerous applications. Researchers and engineers throughout the world are interested in nanofluids because they have the potential to increase the thermal conductivity of typical fluids such as liquid water, ethylene glycol, compressor oil, engine oil, and synthetic oil. Because of their inherent limited heat transfer capacities, these fluids frequently meet restrictions in heat transfer procedures. The incorporation of tiny solid particles, known as tiny particles, into these fluids has the potential to increase their thermal conductivity. The introduction of nanostructures to the base fluid significantly increased both the resultant fluid's and the base fluid's thermal conductivity, as demonstrated by Choi^1^. Buongiorno et al.^2^ investigated the usage of nanofluid in nuclear equipment. Nanofluids were shown to be more profitable and beneficial than base fluids in nuclear power plants. Alamri et al.^3^ deliberate the influence of mass movement throughout a viscoelastic liquid flow on heat conduction and the calming time necessary to obtain a regulated state. Babazadeh et al.^4^ studied a computational method for the mobility of nanomaterials in permeable space. Niazmand et al.^5^ examined the consequence of nanomaterials in the wall of a cylindrical hollow powered by a lamp. Ullah et al.^6^ explored the overall sum of forced thermal conduction in a vertical duct that has been partially heated and is filled with copper nanofluids derived from water that has oxidized. Goyal et al.^7^ explored heat and mass transmission in a micropolar liquid by reducing the layer. Yasmin et al.^8^ go into great depth about the heat transfer and mass caused by curved stretched sheets in MHD flow with electrical wires. Kumar et al.^9^ experimentally investigated the effect and heat transmission properties of a micropolar permeable MHD material with entropy generation and an extended sheet in the second item sliding velocity acting as a heat source and sink. Shamsuddin et al.^10^ investigate both the heat source and sink nonlinear stable, hydromagnetic, micropolar radiation flow. Reference^11^ are looking into the travel qualities of a magneto micropolar liquid with heat micro structural overload sheet in an insulating material under two separate heat boundary circumstances. Numerous researchers have concentrated on nanofluid flows, which are backed by references discovered via investigations^12–22^.

Bioconvection is the movement of low-density microorganisms on the outermost layer of a liquid, which leads to the formation of disordered designs and stability. Because of their swimming behavior, movable microorganisms such as algae tend to collect in the upper layer of the fluid, resulting in a brittle upper area and increased stratification density. Nanomaterial mobility differs from that of motile microorganisms. The interplay between bioconvection and water nanofluids is critical in the setting of microfluidics. Mobile microbe mobility is a critical difficulty in the creation of microfluidic systems. Related devices include bio-galvanic devices and life science colloidal systems. This issue is being researched for a range of thermophysical processes connected to elevated temperature slopes, microbial oil reserves, and the management of sediment sinks for oil and natural gas transportation. Reference^23^ defined bioconvection and investigated polygonal revolving systems in dense Tetrahymena colonies. Then Ref.^24^ discovered significant data regarding the absorption of nanostructures composed of motile bacteria. Reference^25^ explored the Riga-plate thermo-bioconvection of nanofluids flow. Khan et al.^15,16^ demonstrated the Convection flow of the Buongiorno nanofluid design on two stretchy rotating discs. Shafiq et al.^26^ inspected the second-grade advection nanofluid movement with heat and mass transfer impacts, including motile microorganisms. Many researchers and scientists have studied the phenomenon of bioconvection in the following references^20,21,27–30^.

- There has been very little study on bioconvection in the magnetohydrodynamics (MHD) flow of micropolar nanofluid across an oscillating surface under the effect of motile microorganisms, and a non-uniform heat source/sink.
- These gaps in the literature were filled by this study, which employed the shooting method and the shot methodology to mimic the motion of micropolar nanofluids.
- To generate the regulating nonlinear flow equations in the mathematical framework, the stretchable surface needs similarity renovation.
- To match our requirements, we created the appropriate tables and graphs for the well-established parameters.
- When the estimated results are compared to the current data under various limiting situations, they show excellent agreement.

## Physical and mathematical description

### Physical description

**Figure 1:**
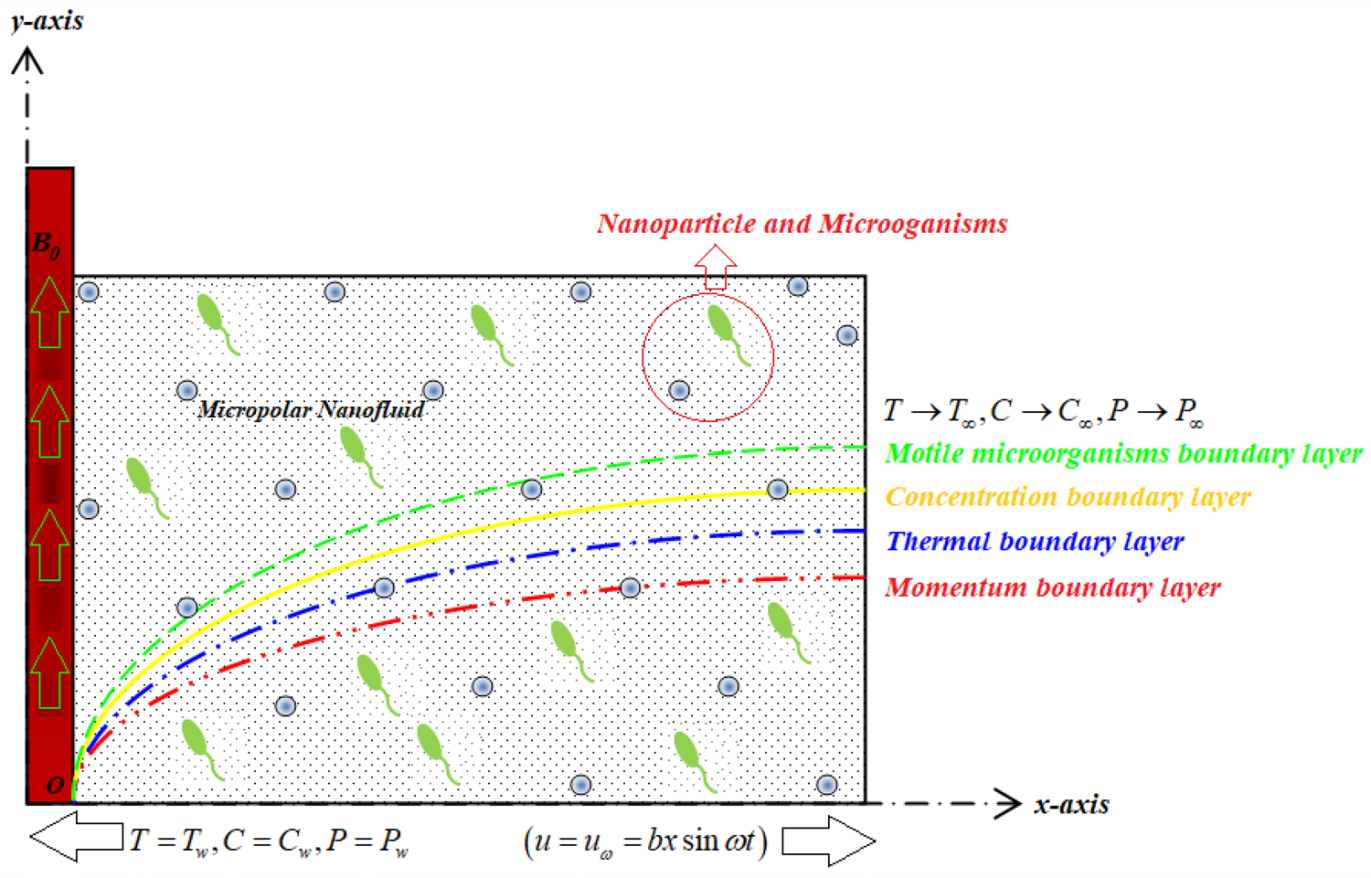
Flow configuration of the current problem.

Consider the 2D Bio-convectional viscoelastic micropolar nano liquid flow with the consequence of thermal conductivity and thermal energy over an oscillating surface (see Fig. 1). Let $\left( {T_{w} } \right)$,$\left( {C_{w} } \right)$ and $\left( {P_{w} } \right)$ are depicts constant temperature, constant concentration, and constant microorganisms. The $\left( {T_{\infty } } \right)$,$\left( {C_{\infty } } \right)$ and $\left( {P_{\infty } } \right)$ are denotes the permitted stream temperature, free stream concentration, and free stream microorganisms divided. The velocity of the fluid is $\left( {u = u_{\omega } = bx\sin \omega t} \right)$ with the magnetic field effects. In consideration of these circumstances, the fundamental equations for viscoelastic micropolar nanofluid flow are as follows:

### Mathematical description

Here $\left( \mu \right)$ is the active viscosity, $\left( {\tilde{p}} \right)$ the pressure, $\left( N \right)$ micro-rotation velocity, $\left( {\mathbf{V}} \right)$ the velocity vector,$\left( {\gamma_{1} } \right)$ the rotation slope, and $\left( {k_{1} } \right)$ the viscosity of a vortex.

The vector forms of governing equation are as^15,16^

1 $$\nabla .{\mathbf{V}} = 0,$$

2 $$\rho_{f} \frac{{d{\mathbf{V}}}}{dt} = - \nabla \tilde{p} + \left( {\mu + k_{1} } \right)\nabla^{2} {\mathbf{V}} + k_{1} \nabla .N,$$

3 $$\rho_{f} j\frac{dN}{{dt}} = \gamma_{1} \nabla \left( {\nabla .N} \right) - \gamma_{1} \nabla \left( {\nabla + N} \right) + k_{1} \nabla .{\mathbf{V}} - 2k_{1} \nabla .N.$$

For the presented velocity profile

4 $${\mathbf{V}}\left[ { = \left\{ {u\left( {x,y,t} \right),\,v\left( {x,y,t} \right),0} \right\}} \right].$$

The governing expressions for viscoelastic micropolar nanomaterials are given below^31,32^

### Equation of continuity

5 $$u_{x} + v_{y} = 0.$$

### Equation of momentum

6 $$\begin{aligned} u_{t} + uu_{x} + vu_{y} & = \left( {v + \frac{{k_{1} }}{{\rho_{f} }}} \right)u_{yy} + \frac{{k_{1} }}{{\rho_{f} }}N_{y} - \frac{{\alpha_{1} }}{{\rho_{f} }}\left[ {u_{tyy} + \partial_{t} \left( {uu_{yy} } \right) + u_{y} v_{yy} + vu_{yyy} } \right] - \frac{{\sigma_{e} B_{0}^{2} }}{{\rho_{f} }}u \\ & \quad + \frac{1}{{\rho_{f} }}\left[ {\left\{ {\left( {1 - C_{f} } \right)\rho_{f} \beta^{*} g\left( {T - T_{\infty } } \right) - \left( {\rho_{p} - \rho_{f} } \right)g\left( {C - C_{\infty } } \right) - \left( {N - N_{\infty } } \right)g\gamma^{**} \left( {\rho_{m} - \rho_{f} } \right)} \right\}} \right]. \\ \end{aligned}$$

### Equation of micro rotation

7 $$N_{t} + uN_{x} + vN_{y} = \frac{\gamma }{{\rho_{f} j}}N_{yy} - \frac{{k_{1} }}{{\rho_{f} j}}\left( {2N + u_{y} } \right).$$

### Equation of Energy

8 $$\begin{aligned} T_{t} + uT_{x} + vT_{y} & = \frac{1}{{\rho c_{p} }}\partial_{z} \left[ {k\left( T \right)T_{z} } \right] + \left( {\alpha_{f} + \frac{{16\sigma_{q} T_{\infty }^{3} }}{{3k^{*} \left( {\rho C} \right)f}}} \right)T_{yy} + \tau_{1} \left[ {D_{B} C_{y} T_{y} + \frac{{D_{T} }}{{T_{\infty } }}\left( {T_{y} } \right)^{2} } \right] \\ & \quad + \frac{1}{{\rho c_{p} }}\left[ {\left( {\frac{{kU_{w} \left( x \right)}}{{\left( {x + y} \right)\upsilon }}} \right)\left( {A^{*} \left( {T_{w} - T_{\infty } } \right)f^{\prime} + B^{*} \left( {T - T_{\infty } } \right)} \right)} \right], \\ \end{aligned}$$

where

9 $$K\left( T \right) = k_{\infty } \left\{ {\left( {1 + \in_{1} \frac{{T - T_{\infty } }}{\Delta T}} \right)} \right\}.$$

### Equation of concentration

10 $$C_{t} + uC_{x} + vC_{y} = \frac{1}{{\rho c_{p} }}\partial_{z} \left[ {D\left( T \right)C_{z} } \right] + D_{B} C_{yy} + \frac{{D_{T} }}{{T_{\infty } }}\left( {T_{yy} } \right),$$

where

11 $$D\left( C \right) = k_{\infty } \left( {1 + \in_{2} \frac{{C - C_{\infty } }}{\Delta C}} \right)$$

### Equation of motile microorganisms

12 $$P_{t} + uP_{x} + vP_{y} = D_{m} \left( {P_{yy} } \right) - \frac{{bW_{c} }}{{\left( {C_{w} - C_{\infty } } \right)}}\left[ {\partial y\left( {PC_{y} } \right)} \right].$$

### Boundary conditions

The boundary conditions are

13 $$\left. \begin{gathered} u = u_{w} + l\left[ {\left( {\mu + k} \right)u_{y} + kN} \right],\,v = 0,N = 0, - kT_{y} = h_{f} \left( {T_{w} - T} \right), \hfill \\ D_{B} C_{y} + \frac{{D_{T} }}{{T_{\infty } }}T_{y} = 0,P = P_{\omega } \,at\,y = 0, \hfill \\ u \to 0,u^{\prime} \to 0,v \to 0,N \to 0,T \to T_{\infty } ,C \to C_{\infty } ,P \to P_{\infty } \,at\,y \to \infty . \hfill \\ \end{gathered} \right\}.$$

Also, with the melting phenomenon

14 $$- k\left. {\left( {T_{y} } \right)} \right|_{\,\,y = 0} = \rho \left[ {\lambda + \left( {T_{m} - T_{0} } \right)c_{s} } \right].$$

### Similarity analysis

The similarity transformations are^33–37^

15 $$\left. \begin{gathered} \zeta \left[ { = \sqrt{\frac{a}{v}} y} \right],\,u\left[ { = axf^{\prime}\left( {\zeta ,\tau } \right)} \right],v\left[ { = - \sqrt {va} f\left( {\zeta ,\tau } \right)} \right],N\left[ { = ax\left( \frac{a}{v} \right)^{\frac{1}{2}} g\left( {\zeta ,\tau } \right)} \right], \hfill \\ \tau \left[ { = t_{\omega } } \right]. \hfill \\ \theta \left( {\zeta ,\tau } \right)\left[ { = \frac{{T - T_{\infty } }}{{T_{\omega } - T_{\infty } }}} \right],\,\,\,\,\,\,\,\,\,\,\phi \left( {\zeta ,\tau } \right)\left[ { = \frac{{C - C_{\infty } }}{{C_{\omega } - C_{\infty } }}} \right],\,\,\,\,\,\,\,\,\,\,\,\,\chi \left( {\zeta ,\tau } \right)\left[ { = \frac{{P - P_{\infty } }}{{P_{\omega } - P_{\infty } }}} \right]. \hfill \\ \end{gathered} \right\}.$$

### Dimensionless governing equations

Substitution of the above equations can result in the following dimensionless equations

16 $$\left( {1 + K} \right)f^{\prime\prime\prime} - Sf^{\prime} + Kg^{\prime} + ff^{\prime\prime} - \left( {f^{\prime}} \right)^{2} - Mf^{\prime} - k\left[ \begin{gathered} Sf^{\prime\prime\prime} + 2f^{\prime}f^{\prime\prime\prime} \hfill \\ - \left( {f^{\prime\prime}} \right)^{2} - ff^{iv} \hfill \\ \end{gathered} \right] - \lambda \left( {\theta - Nr\phi - Nc\chi } \right) = 0,$$

17 $$\left( {1 + \frac{K}{2}} \right)g^{\prime\prime} - K\left( {2g + f^{\prime\prime}} \right) - Sg - f^{\prime}g + g^{\prime}f = 0,$$

18 $$\frac{1}{\Pr }\left( {1 + \in_{1} Rd\left( {1 + \left( {\theta_{w} - 1} \right)\theta^{3} } \right)} \right)\theta^{\prime\prime} + \in_{1} \theta^{{\prime}{2}} - S\theta + Nb\theta^{\prime}\phi^{\prime} + Nt\theta^{{\prime}{2}} + f\theta^{\prime} + \left( {A^{*} f^{\prime} + B^{*} \theta } \right) = 0,$$

19 $$\frac{1}{Le\Pr }\left( {1 + \in_{2} } \right)\phi^{\prime\prime} + \in_{2} \phi^{{\prime}{2}} + \frac{Nt}{{Nb}}\theta^{\prime\prime} - S\phi + f\phi^{\prime} = 0,$$

20 $$\chi ^{\prime\prime} + Lbf\chi ^{\prime} - Pe\left( {\phi ^{\prime\prime}\left( {\chi + \delta_{1} } \right) + \chi ^{\prime}\phi ^{\prime}} \right) = 0.$$

With

21 $$\left. \begin{gathered} f^{\prime}\left( {0,\tau } \right) = 1 + \beta \left( {1 + \frac{\alpha }{2}} \right)f^{\prime\prime}\left( {0,\tau } \right),\,\,\,\,\,\,\,\,\,\,\,\,\,\,\,\,\,\,f\left( {0,\tau } \right) = 0, \hfill \\ g\left( {0,\tau } \right) = 0,\,\,\,\,\,\,\,\,\,\,\,\,\,\,\,\,\,\,\,\,\,\,\,\,\,\,\,\,\,\,\,\theta^{\prime}\left( {0,\tau } \right) = - Bi\left( {1 - \theta \left( {0,\tau } \right)} \right), \hfill \\ Nb\phi^{\prime}\left( {0,\tau } \right) + Nt\theta^{\prime}\left( {0,\tau } \right) = 0,\,\,\,\,\,\,\,\,\,\,\,\,\,\,\,\,\,\,\,\,\,\,\,\,\,\,\,\,\chi \left( {0,\tau } \right) = 1, \hfill \\ f^{\prime}\left( {0,\tau } \right) \to 0,\,\,\,\,\,\,\,\,\,\,\,\,\,\,\,\,\,\,\,\,\,\,\,\,\,\,\,\,\,\,\,\,\,\,\,\,\,\,\,\,\,\,\,\,\,\,\,\,\,\,\,\,\,\,\,\,f^{\prime\prime}\left( {0,\tau } \right) \to 0, \hfill \\ g\left( {0,\tau } \right) \to 0,\,\,\,\,\,\,\,\,\,\,\,\,\,\,\,\,\,\,\,\,\,\,\,\,\,\,\,\,\,\,\,\,\,\,\,\,\,\,\,\,\,\,\,\,\,\,\,\,\,\,\,\,\,\,\,\,\,\,\,\,\theta \left( {0,\tau } \right) \to 0, \hfill \\ \phi \left( {0,\tau } \right) \to 0,\,\,\,\,\,\,\,\,\,\,\,\,\,\,\,\,\,\,\,\,\,\,\,\,\,\,\,\,\,\,\,\,\,\,\,\,\,\,\,\,\,\,\,\,\,\,\,\,\,\,\,\,\,\,\,\,\,\,\,\,\chi \left( {0,\tau } \right) \to 0. \hfill \\ \end{gathered} \right\}.$$

The melting phenomenon has no dimensions.

22 $$Me\theta \left( {0,\tau } \right) + \Pr f\left( {0,\tau } \right) = 0.$$

### Physical flow parameters

**Table:** 

Mathematical values	Name of parameter
$k\left( { = \frac{{ak_{0} }}{{v\rho_{f} }}} \right)$	Viscoelastic parameter
$M\left( { = \sqrt {\frac{{\sigma_{e} B_{0}^{2} }}{{\rho_{f} a}}} } \right)$	Hartmann number
$$Nc\left( { = \frac{{\gamma^{**} \left( {\rho_{m} - \rho_{f} } \right)\left( {N_{w} - N_{\infty } } \right)}}{{\left( {1 - C_{\infty } } \right)\left( {T_{w} - \tilde{T}_{\infty } } \right)\beta }}} \right)$$	Bioconvection Rayleigh number
$\lambda \left( { = \frac{{\beta^{*} g\left( {1 - C_{\infty } } \right)\left( {T_{w} - T_{\infty } } \right)}}{{\left( {m + 1} \right)u_{e}^{2} }}} \right)$	Mixed convection parameter
$K\left( { = \frac{{k_{1} }}{{\rho_{f} \upsilon }}} \right)$	Vertex viscosity constant
$Nt\left( { = \frac{{ED_{T} \left( {T_{w} - T_{\infty } } \right)}}{{T_{\infty } \alpha }}} \right)$	Thermophoresis parameter
$Nr\left( { = \frac{{\left( {\rho_{p} - \rho_{f} } \right)\left( {C_{w} - C_{\infty } } \right)}}{{\left( {1 - C_{\infty } } \right)\left( {T_{w} - T_{\infty } } \right)\beta^{*} }}} \right)$	Buoyancy ratio parameter
$Lb\left( { = \frac{\nu }{{D_{m} }}} \right)$	Bioconvection Lewis number
$Pe\left( { = \frac{{bW_{c} }}{{D_{m} }}} \right)$	Peclet number
$\Pr \left( { = \frac{v}{\alpha }} \right)$	Prandtl number
$Me\left( { = \frac{{c_{p} \left( {T_{\infty } - T_{m} } \right)}}{{\lambda + c_{s} \left( {T_{m} - T_{0} } \right)}}} \right)$	Melting parameter
$Bi\left( { = \frac{{h_{f} }}{k}\sqrt {\frac{\nu }{c}} } \right)$	Biot number
$\delta_{1} \left( { = \frac{{N_{\infty } }}{{N_{w} - N_{\infty } }}} \right)$	Microorganism difference parameter
$\left( {S = \frac{b}{a}} \right)$	Unsteady parameter
$Rd\left( { = \frac{{16\sigma^{*} T_{\infty }^{3} }}{{3kk^{*} }}} \right)$	Radiation parameter
$Le\left( { = \frac{\alpha }{{D_{B} }}} \right)$	Lewis number
$\alpha \left( { = \frac{k}{\mu }} \right)$	Angular micropolar parameter
$Nb\left( { = \frac{{ED_{B} \left( {C_{w} - C_{\infty } } \right)}}{\alpha }} \right)$	Brownian motion parameter
$\theta_{w} \left( { = \frac{{T_{w} }}{{T_{\infty } }}} \right)$	Temperature ratio parameter
$\beta \left( { = l\sqrt {\mu \rho \psi } } \right)$	Velocity slip parameter

### Physical quantities

Active Sherwood number $\phi^{\prime}\left( 0 \right)$, Nusselt number $- \theta^{\prime}\left( 0 \right)$, and Microorganism density number $\chi^{\prime}\left( 0 \right)$ compared to heat and mass transfer rate can be proposed as:

23 $$\frac{{Nu_{x} }}{{\sqrt {{\text{Re}}_{x} } }} = - \theta^{\prime}\left( {0,\tau } \right),\frac{{Sh_{x} }}{{\sqrt {{\text{Re}}_{x} } }} = - \phi^{\prime}\left( {0,\tau } \right),\frac{{Sn_{x} }}{{\sqrt {{\text{Re}}_{x} } }} = - \chi \left( {0,\tau } \right).$$

Here the local Reynolds number is ${\text{Re}}_{x} = \frac{{u_{\omega } x}}{v}$.

## Numerical method

The controlling ODEs (16–20) for the movement and heat and mass transport of cross-nanofluid with the consequences of activation and motile microorganisms are numerically controlled with boundary conditions (21–22) by the employing the bvp4c scheme^34–37^ in MATLAB. To solve the issue numerically, appropriate transformation similarity techniques and shooting techniques are employed. The first step is to convert the governing structure's flow equation to an ODE of the first order.

Let

24 $$\left. \begin{gathered} f = h_{1} ,f^{\prime} = h_{2} ,f^{\prime\prime} = h_{3} ,f^{\prime\prime\prime} = h_{4} ,f^{iv} = h^{\prime}_{4} , \hfill \\ g = h_{5} ,g^{\prime} = h_{6} ,g^{\prime\prime} = h^{\prime}_{6} \hfill \\ \theta = h_{7} ,\theta^{\prime} = h_{8} ,\theta^{\prime\prime} = h^{\prime}_{8} \hfill \\ \phi = h_{9} ,\phi^{\prime} = h_{10} ,\phi^{\prime\prime} = h^{\prime}_{10} \hfill \\ \chi = h_{11} ,\chi^{\prime} = h_{12} ,\chi^{\prime\prime} = h^{\prime}_{12} \hfill \\ \end{gathered} \right\},$$

25 $$h^{\prime}_{4} = \frac{\begin{gathered} - \left( {1 + K} \right)h_{4} + Sh_{2} - Kh_{6} - h_{1} h_{3} + \left( {h_{2} } \right)^{2} + Mh_{2} + k\left[ {Sh_{4} + 2h_{2} h_{4} - \left( {h_{3} } \right)^{2} } \right] \hfill \\ + \lambda \left( {h_{7} - Nrh_{9} - Nch_{11} } \right) \hfill \\ \end{gathered} }{{kh_{1} }},$$

26 $$h^{\prime}_{6} = \frac{{K\left( {2h_{5} + h_{3} } \right) + Sh_{5} + h_{2} h_{5} - h_{6} h_{1} }}{{\left( {1 + \frac{K}{2}} \right)}},$$

27 $$h^{\prime}_{8} = \frac{{\Pr \left( { - \in_{1} h_{8}^{2} + Sh_{7} - Nbh_{8} h_{10} - Nth_{8}^{2} - h_{1} h_{8} } \right) - \Pr \left( {A^{*} h_{2} + B^{*} h_{7} } \right)}}{{\left( {1 + \in_{1} Rd\left( {1 + \left( {\theta_{w} - 1} \right)h_{7}^{3} } \right)} \right)}},$$

28 $$h^{\prime}_{10} = \frac{{ - \in_{2} h_{10}^{2} - \Pr Le\frac{Nt}{{Nb}}h^{\prime}_{8} + \Pr LeSh_{9} - \Pr Leh_{1} h_{10} ,}}{{\left( {1 + \in_{2} } \right)}},$$

29 $$h^{\prime}_{12} = - Lbh_{1} h_{12} + Pe\left( {h^{\prime}_{10} \left( {h_{11} + \delta_{1} } \right) + h_{12} h_{10} } \right).$$

With

30 $$\left. \begin{gathered} h_{2} \left( {0,\tau } \right) = 1 + \beta \left( {1 + \frac{\alpha }{2}} \right)h_{3} \left( {0,\tau } \right),\,\,\,\,\,\,\,h_{1} \left( {0,\tau } \right) = 0,h_{5} \left( {0,\tau } \right) = 0, \hfill \\ h_{8} \left( {0,\tau } \right) = - Bi\left( {1 - h_{7} \left( {0,\tau } \right)} \right), \hfill \\ Nbh_{10} \left( {0,\tau } \right) + Nth_{8} \left( {0,\tau } \right) = 0,\,\,\,\,\,\,\,\,\,\,\,\,\,\,\,\,\,\,\,\,\,\,\,\,\,\,\,\,\,\,\,\,\,\,\,\,\,\,\,h_{11} \left( {0,\tau } \right) = 1, \hfill \\ h_{2} \left( {0,\tau } \right) \to 0,\,\,\,\,\,\,\,\,\,\,\,\,\,\,\,\,\,\,\,\,\,\,\,\,\,\,\,\,\,\,\,\,\,\,\,\,\,\,\,\,\,\,\,\,\,\,\,\,\,\,\,\,\,\,\,\,\,\,\,\,\,\,\,\,\,\,\,\,\,\,\,h_{3} \left( {0,\tau } \right) \to 0,\, \hfill \\ h_{5} \left( {0,\tau } \right) \to 0,\,\,\,\,\,\,\,\,\,\,\,\,\,\,\,\,\,\,\,\,\,\,\,\,\,\,\,\,\,\,\,\,\,\,\,\,\,\,\,\,\,\,\,\,\,\,\,\,\,\,\,\,\,\,\,\,\,\,\,\,\,\,\,\,\,\,\,\,\,\,\,h_{7} \left( {0,\tau } \right) \to 0,\, \hfill \\ h_{9} \left( {0,\tau } \right) \to 0,\,\,\,\,\,\,\,\,\,\,\,\,\,\,\,\,\,\,\,\,\,\,\,\,\,\,\,\,\,\,\,\,\,\,\,\,\,\,\,\,\,\,\,\,\,\,\,\,\,\,\,\,\,\,\,\,\,\,\,\,\,\,\,\,\,\,\,\,\,\,h_{11} \left( {0,\tau } \right) \to 0. \hfill \\ \end{gathered} \right\},$$

31 $$Meh_{7} \left( {0,\tau } \right) + \Pr h_{1} \left( {0,\tau } \right) = 0.$$

### Code validation

Table 1 discloses the validation of current results with published results. Here good agreement is seen between current results and previously published results.

**Table 1:** Validation of computation for $f_{\zeta \zeta } \left( {0,\tau } \right)$ at $k = 0,\lambda = 0 = Nr = Nc = Pe = Le = 0$, $A^{*} = 0 = B^{*} ,S = 0,\tau = 8.5\pi$ for various amounts of $M$.

$M$	*K* = 0.0		*K* = 0.025	
	El-kabeir^38^	Current results	El-kabeir^38^	Current results
0.0	1.2325	1.2325	1.2629	1.26296
0.2	1.2487	1.2485	1.2793	1.27925
0.4	1.2953	1.2953	1.3272	1.32727
0.6	1.3698	1.3698	1.4036	1.40355
0.8	1.4679	1.4679	1.5042	1.50421
1.0	1.5853	1.5853	1.6247	1.62472

### Tabular values

Tables 2, 3, 4 are depicted the numerical inspection of $- f^{\prime\prime}\left( 0 \right)$, $- g^{\prime\prime}\left( 0 \right)$, $- \theta^{\prime}\left( 0 \right)$, $- \phi^{\prime}\left( 0 \right)$ and $\chi^{\prime}\left( 0 \right)$. Table 2 depicts that the skin friction coefficients and micro rotation profile are diminished for the melting parameter and material parameter while the magnetic parameter is boosted up. Tables 3 and 4 are suggested as a way to examine the local Sherwood number and Nusselt number using physical flow characteristics. Local Sherwood and Nusselt numbers are increasing in this case for the material parameter while melting and magnetic parameters are decreasing. Table 5 shows that the microorganism density number for the Peclet number and the bioconvection Lewis number is increasing.

**Table 2:** Numerical values of $- f^{\prime\prime}\left( 0 \right)$ and $- g^{\prime\prime}\left( 0 \right)$ via physical parameters.

$M$	$Me$	$\lambda$	$K$	$\alpha$	$\beta$	$Nr$	$Nc$	$- f^{\prime\prime}\left( 0 \right)$	$- g^{\prime\prime}\left( 0 \right)$
0.2	0.4	0.1	0.1	0.1	0.4	0.1	0.1	0.6061	0.1877
0.8								0.8053	0.2515
1.6								0.9943	0.3085
0.1	0.5							0.5524	0.1652
	0.7							0.5127	0.1460
	0.9							0.4726	0.1276
		0.2						0.5374	0.1662
		0.4						0.5477	0.1710
		0.7						0.5686	0.1906
			0.2					0.7961	0.2511
			0.4					0.7671	0.2210
			0.6					0.7412	0.1970
				0.2				0.4727	0.1386
				0.5				0.3177	0.0784
				1.0				0.2403	0.0504
					0.1			0.5524	0.0121
					0.5			0.5363	0.2242
					0.9			0.5211	0.4483
						0.2		0.7959	0.2509
						1.0		0.7921	0.2474
						2.0		0.7904	0.2454
							0.2	0.8009	0.2524
							1.0	0.8408	0.2627
							2.0	0.8909	0.2745

**Table 3:** Numerical values $- \theta^{\prime}\left( 0 \right)$ via physical parameters.

$M$	$Me$	$\lambda$	$K$	$\alpha$	$\beta$	$Bi$	$Rd$	$A^{*}$	$B^{*}$	$- \theta^{\prime}\left( 0 \right)$
0.2	0.4	0.1	0.1	0.1	0.4	0.2	0.5	0.1	0.1	0.5130
0.8										0.4751
1.6										0.4572
0.1	0.5									0.5044
	0.7									0.4771
	0.9									0.4500
	0.4	0.2								0.5464
		0.4								0.5378
		0.7								0.5285
			0.2							0.4896
			0.4							0.4920
			0.6							0.4943
				0.2						0.4727
				0.5						0.4569
				1.0						0.4486
					0.1					0.4778
					0.5					0.4795
					0.9					0.4811
						0.4				0.2600
						1.4				0.4690
						2.4				0.5394
							0.1			0.5901
							0.7			0.4824
							1.4			0.4131
								02		0.1837
								0.4		0.1264
								0.6		0.0476
									02	0.1648
									0.4	0.1599
									0.6	0.1202

**Table 4:** Numerical values $\phi^{\prime}\left( 0 \right)$ via physical parameters.

$M$	$Me$	$\lambda$	$K$	$\alpha$	$\beta$	$Bi$	$Rd$	$Le$	$\phi^{\prime}\left( 0 \right)$
0.2	0.4	0.1	0.1	0.1	0.4	0.2	0.5	0.1	0.7695
0.8									0.7276
1.6									0.6858
0.1	0.5								0.7566
	0.7								0.7156
	0.9								0.6749
		0.2							0.3567
		0.4							0.2358
		0.7							0.1357
			0.2						0.7341
			0.4						0.7380
			0.6						0.7414
				0.2					0.7092
				0.5					0.6854
				1.0					0.6727
					0.1				0.7168
					0.5				0.7192
					0.9				0.7216
						0.4			0.3899
						1.4			0.7044
						2.4			0.8091
							0.1		0.8851
							0.7		0.7236
							1.4		0.6206
								02	0.7812
								0.4	0.7736
								0.6	0.7764

**Table 5:** Numerical values $\chi^{\prime}\left( 0 \right)$ via physical parameters.

$M$	$Me$	$\lambda$	$K$	$\alpha$	$\beta$	$Pe$	$Lb$	$\chi^{\prime}\left( 0 \right)$
0.2	0.4	0.1	0.1	0.1	0.4	0.2	0.5	0.5723
0.8								0.5191
1.6								0.4690
0.1	0.5							0.5588
	0.7							0.5117
	0.9							0.4647
		0.2						0.4571
		0.4						0.3511
		0.7						0.3456
			0.2					0.5288
			0.4					0.5343
			0.6					0.5393
				0.2				0.8989
				0.5				0.7519
				1.0				0.7277
					0.1			0.8137
					0.5			0.8178
					0.9			0.8217
						0.4		0.6501
						1.4		1.2106
						2.4		1.6502
							0.1	0.6390
							0.7	0.8510
							1.4	0.9992

## Results and discussion

**Figure 2:**
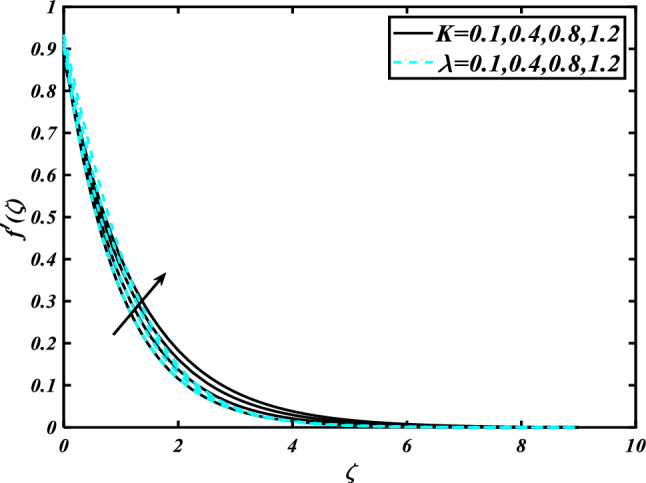
Inspirations of $f^{\prime}$ through $K\& \lambda$.

**Figure 3:**
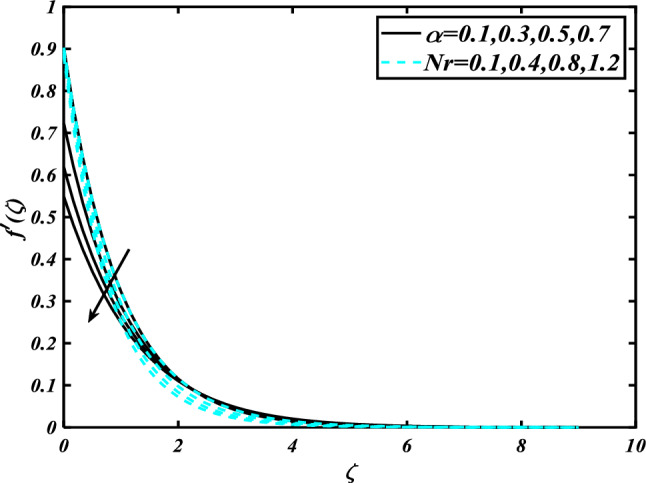
Inspirations of $f^{\prime}$ through $\alpha \& Nr$.

**Figure 4:**
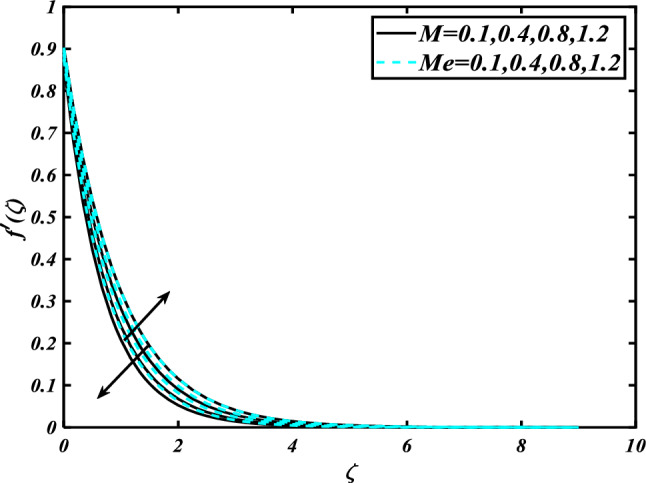
Inspirations of $f^{\prime}$ through $M\& Me$.

**Figure 5:**
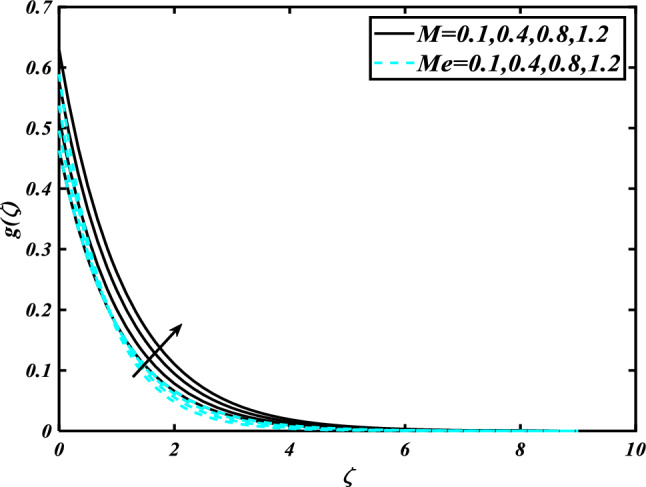
Inspirations of $g$ through $M\& Me$.

**Figure 6:**
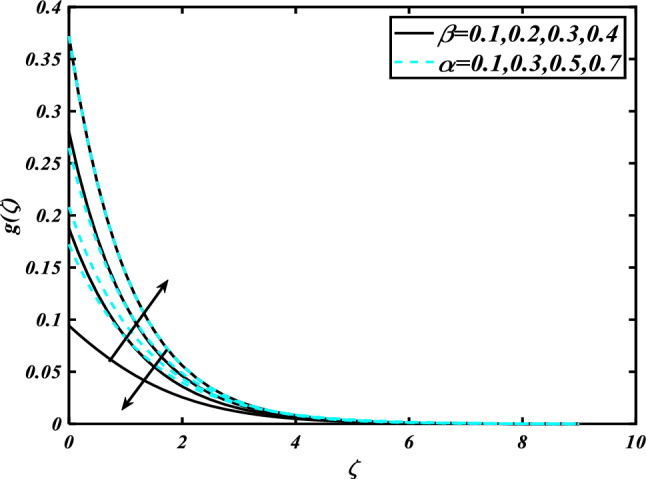
Inspirations of $g$ through $\beta \& \alpha$.

**Figure 7:**
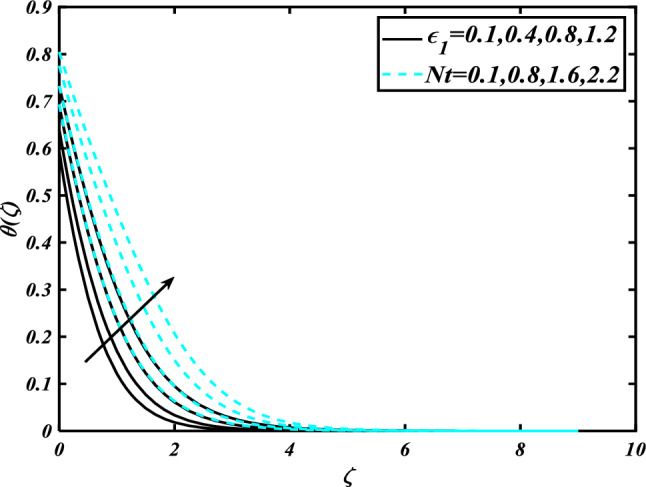
Inspirations of $\theta$ through $\in_{1} \& Nt$.

**Figure 8:**
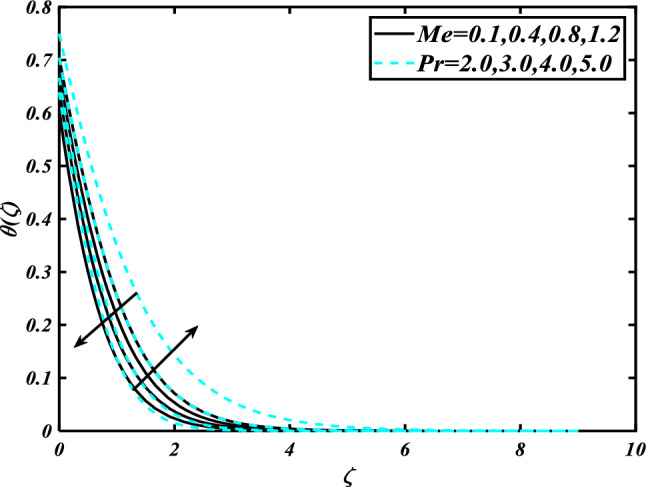
Inspirations of $\theta$ through $Me\& \Pr$.

**Figure 9:**
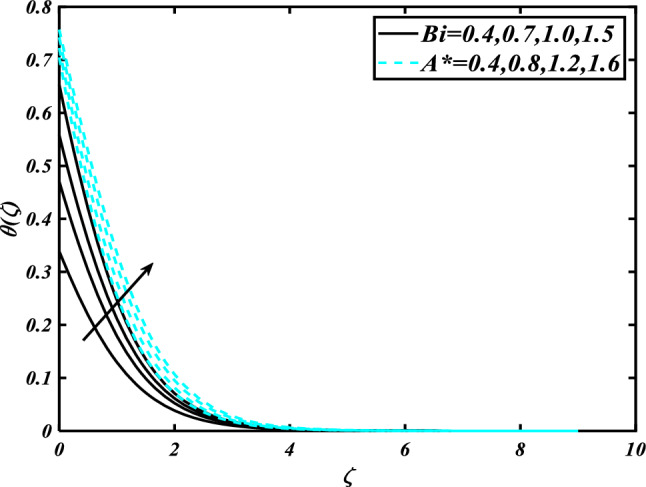
Inspirations of $\theta$ through $Bi\& A^{*}$.

**Figure 10:**
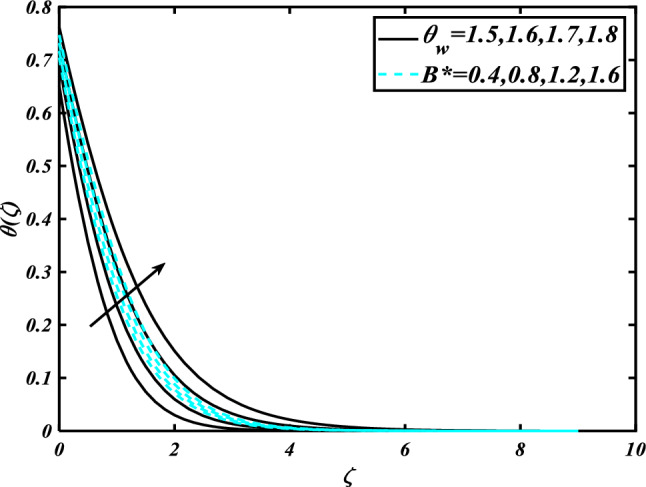
Inspirations of $\theta$ through $\theta_{w} \& B^{*}$.

This section highlights the importance of controlling parameters on the velocity profile, micro rotation profile, thermal profile, concentration profile, and microorganism profile. Figure 2 is demonstrated to expose the aspects of the mixed convection parameter $\left( \lambda \right)$ and vertex viscosity constant $\left( K \right)$ on the velocity distribution profile $\left( {f^{\prime}} \right)$. The velocity profile boomed up with higher estimations of both variables mixed convection parameter $\left( \lambda \right)$ and vertex viscosity constant $\left( K \right)$. Figure 3 expressed the results of the bouncy ratio parameter $\left( {Nr} \right)$ and $\left( \alpha \right)$ over the velocity distribution profile $\left( {f^{\prime}} \right)$. The velocity field $\left( {f^{\prime}} \right)$ is decreased by increasing the variation of both parameters bouncy ratio parameter $\left( {Nr} \right)$ and $\left( \alpha \right)$. To reveal the aspects of the magnetic parameter $\left( M \right)$ and melting parameter $\left( {Me} \right)$ via velocity profile $\left( {f^{\prime}} \right)$ in Fig. 4. From the fig, it is detected that the velocity distribution profile $\left( {f^{\prime}} \right)$ is increased for the higher values of the melting parameter $\left( {Me} \right)$ while depressed for the value of the magnetic parameter $\left( M \right)$. To reveal the consequences of the magnetic parameter $\left( M \right)$ and melting parameter $\left( {Me} \right)$ passing through $\left( g \right)$ is illustrated in Fig. 5. From the arcs, it is detected that the swelling valuations of the magnetic parameter $\left( M \right)$ and melting parameter $\left( {Me} \right)$ enhanced the micro rotation profile $\left( g \right)$. As a resistive force, the Lorentz force slows the velocity of fluid particles inside the flow domain, changing the physical properties of these events. The boundary layer thickens more when the circumstance is viscoelastic. The significance of $\left( \alpha \right)$ and velocity slip parameter $\left( \beta \right)$ via the velocity profile $g$ is featured in Fig. 6**.** The outcome shows that the micro rotation profile $\left( g \right)$ through being rising variation $\left( \alpha \right)$ and declined for higher velocity slip parameter $\left( \beta \right)$. The consequence of the thermal conductivity parameter $\left( { \in_{1} } \right)$ and thermophoresis parameter $\left( {Nt} \right)$ against the temperature profile $\left( \theta \right)$ is illustrated in Fig. 7**.** The outcomes demonstrate that the temperature profile $\left( \theta \right)$ is enhanced by mounting for the thermophoresis parameter $\left( {Nt} \right)$ and the thermal conductivity parameter $\left( { \in_{1} } \right)$. Figure 8 shows the impact of the Prandtl number $\left( {\Pr } \right)$ and melting parameter $\left( {Me} \right)$ against the temperature profile $\left( \theta \right)$ is articulated**.** The temperature distribution profile $\left( \theta \right)$ intensified by increasing variation of melting parameter $\left( {Me} \right)$ and declined for the Prandtl number $\left( {\Pr } \right)$. The effect on the temperature concentration profile $\left( \theta \right)$ for Biot number $\left( {Bi} \right)$ and space-dependent heat generation/absorption $\left( {A^{*} } \right)$ is shown in Fig. 9. This graph indicates the rising Biot number $\left( {Bi} \right)$ and space-dependent heat generation/absorption $\left( {A^{*} } \right)$ assessed the temperature profile $\left( \theta \right)$. The Biot number is described as the ratio of a body internal radiative heat resistance to its exterior heat transfer resistance. As a consequence, a negligible Biot number implies fewer barriers to heat transmission and, consequently, low-temperature gradients inside the body. The performance of the temperature-dependent heat generation/absorption $\left( {B^{*} } \right)$ and temperature ratio parameter $\left( {\theta_{w} } \right)$ against the temperature profile $\left( \theta \right)$ is revealed in Fig. 10. The temperature concentration profile $\theta$ is enhanced for the escalating environment of temperature-dependent heat generation/absorption $\left( {B^{*} } \right)$ and temperature ratio parameter $\left( {\theta_{w} } \right)$. Physically, the radiative flux accelerates the polymeric movement even though the current radiation factor is increased, adding thermal energy to the process. This temperature causes the boundary layer to thicken.

**Figure 11:**
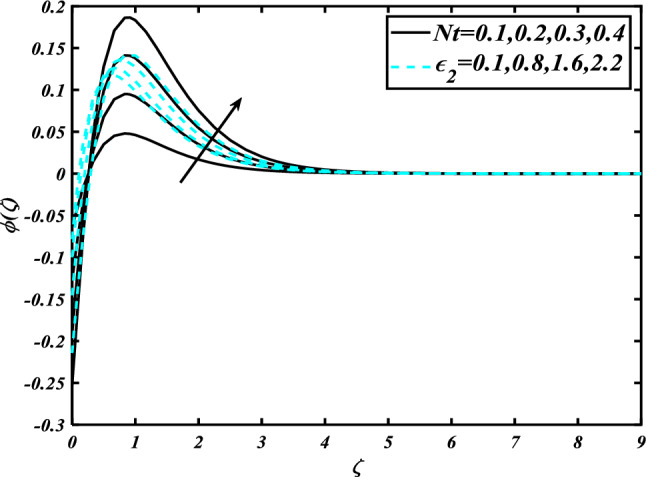
Inspirations of $\phi$ through $Nt\& \in_{2}$.

**Figure 12:**
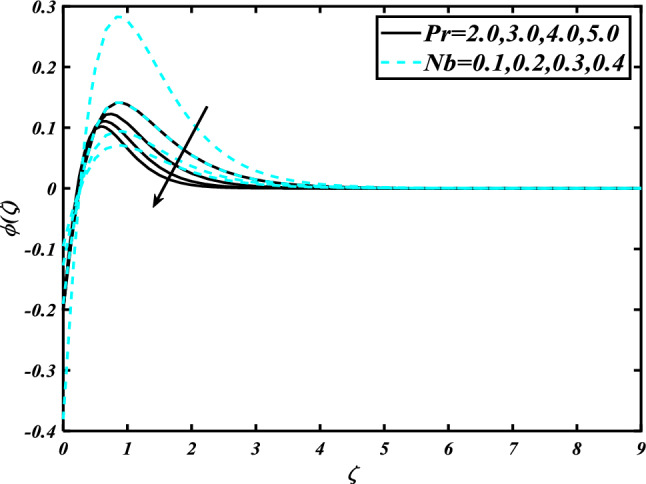
Inspirations of $\phi$ through $\Pr \& Nb$.

**Figure 13:**
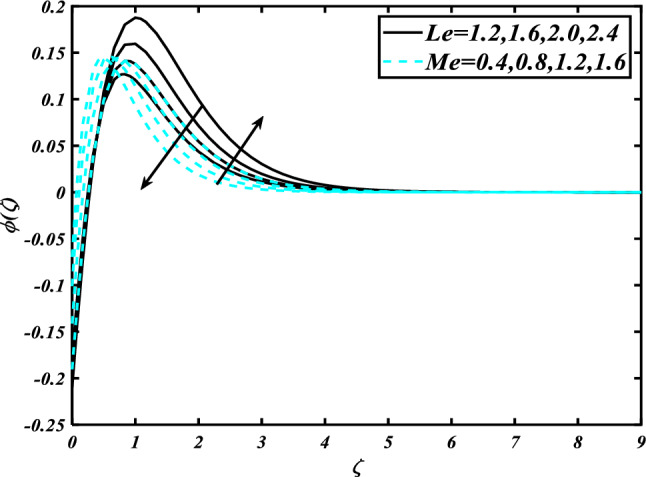
Inspirations of $\phi$ through $Le\& Me$.

**Figure 14:**
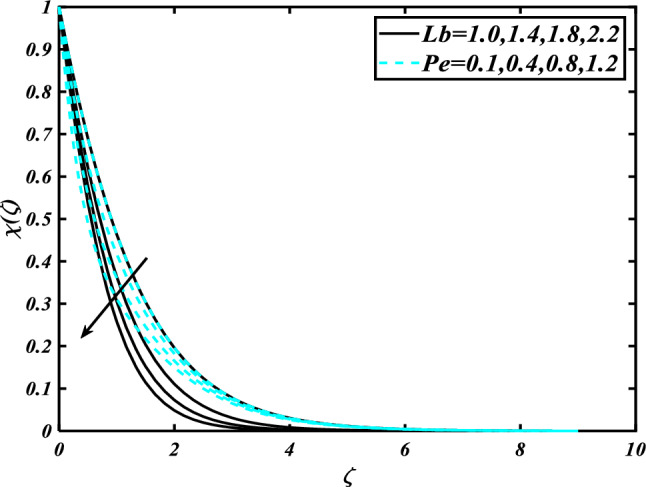
Inspirations of $\chi$ through $Lb\& Pe$.

**Figure 15:**
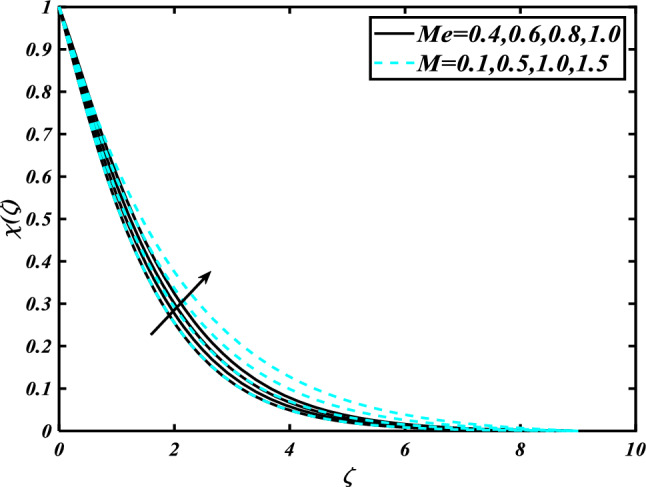
Inspirations of $\chi$ through $Me\& M$.

The nature of the thermophoresis parameter $\left( {Nt} \right)$ and concentration conductivity parameter $\left( { \in_{2} } \right)$ nanoparticles volume fraction is shown in Fig. 11**.** The escalating deviations of the thermophoresis parameter $\left( {Nt} \right)$ and concentration conductivity parameter $\left( { \in_{2} } \right)$ advanced the concentration profile $\left( \phi \right)$. A variety of physical parameters involving heat transfer are commonly used to characterize the thermophoresis phenomenon. This phenomenon occurs as a result of the positive impact of migrating fluid particles accidentally moving in the path of low-temperature zones owing to heat exchange. As a result of the transmission of such moving particles, the heat of the nanomaterials increases with time. Inspect the concentration profile $\left( \phi \right)$ against the Prandtl number $\left( {\Pr } \right)$ which $\left( {Nb} \right)$ is depicted in Fig. 12. Consequently, it can seem that expansion in the concentration profile $\left( \phi \right)$ reduced the $\left( {\Pr } \right)$ and Brownian motion parameter $\left( {Nb} \right)$. The Prandtl number has a physical basis in its description as the ratio of thermal diffusivity to momentum diffusivity. It measures the relative efficiency of momentum and heat transfer by diffusion in the velocity and thermal boundary layers. Inspect the concentration profile $\left( \phi \right)$ against the Lewis number $\left( {Le} \right)$ and the melting parameter $\left( {Me} \right)$ is adorned in Fig. 13. Subsequently, it can seem that by development in the melting parameter $\left( {Me} \right)$, the concentration profile $\left( \phi \right)$ enhanced while it reduces the Lewis number $\left( {Le} \right)$. Figure 14 is apprehended to expose aspects of $\left( {Lb} \right)$ and Peclet number via $\chi$. It is apparent that from the apprehended sketch of flow the microorganism’s profile $\chi$ decays for a higher Peclet number $\left( {Pe} \right)$ and Bioconvection Lewis number $\left( {Lb} \right)$. The percentage of heat-to-mass diffusivity is what defines the Lewis number, which has no dimensions. It is used to explain fluid flows that simultaneously entail the transfer of heat and mass. Figure 15 shows the results of the magnetic parameter $\left( M \right)$ and melting parameter $\left( {Me} \right)$ for the motile microorganisms of nanomaterials nearby. The cumulative disparities of the $\left( M \right)$ and $\left( {Me} \right)$ boomed the microorganism profile $\chi$.

## Conclusions

The presented work aims to observe the bioconvection flow of nano-sized viscoelastic-micropolar liquid with irregular heat source and sink, motile microorganisms by the stretched sheet. Thermal conductivity and thermal radiation are also discussed. This structure has a very efficient application of biomedical, manufacturing, drug, and heating–cooling processes.

- The material parameter causes an increase in the fluid's velocity profile while the bouncy ratio parameter causes a decrease.
- The micro rotation profile enhanced for larger melting parameters while declined for $\beta$.
- The temperature profile is miserable for the Prandtl number while raises with the Biot number and temperature ratio parameter.
- The concentration profile increases with the melting parameter and decreases with the Brownian motion parameter.
- The profile of the microorganism is lowered for the Peclet and bioconvective Lewis number, but it increases for the magnetic parameter.

## Data Availability

The datasets used and analyzed during the current study are available from the corresponding author upon reasonable request.
